# Impact of early rise of intraocular pressure on visual outcome following diabetic vitrectomy

**DOI:** 10.4103/0301-4738.73724

**Published:** 2011

**Authors:** Yog Raj Sharma, Archna Pruthi, Raj Vardhan Azad, Atul Kumar, Rashim Mannan

**Affiliations:** Department of Ophthalmology, Dr. Rajendra Prasad Centre for Ophthalmic Sciences, All India Institute of Medical Sciences, New Delhi, India

**Keywords:** Diabetic retinopathy, intraocular pressure, vitrectomy

## Abstract

**Objective:**

The objective was to study the incidence and risk factors for an early rise in intraocular pressure (IOP) following pars plana vitrectomy (PPV) for proliferative diabetic retinopathy (PDR) and to correlate its impact on visual outcome.

**Materials and Methods:**

This was a longitudinal prospective study. IOP and best corrected visual acuity (BCVA) for 73 cases of PDR (52 males and 21 females) who underwent PPV were recorded at day 1, week 1, and months 1, 3, and 6. Risk factors for the early IOP rise, defined as IOP ≥ 30 mmHg at day 1, were evaluated using cross-tabulation and the *t*-test.

**Results:**

Mean IOP at day 1 was 21.8 ± 9.8 mmHg with 15 cases (20.5%) having an early rise in IOP. Risk factors for the early IOP rise included intraoperative fibrovascular frond removal (*P* = 0.003), lens removal (*P* = 0.043), and intraoperative vitreous bleed (*P* = 0.008). The early rise in IOP was also associated with consistently raised IOP (*P* = 0.02), defined as IOP > 21 mmHg during first three consecutive follow-up visits. Further, difference in BCVA at 6 months among the two groups, i.e., with and without an early IOP rise was statistically significant (3.11 ± 1.52 logMAR vs. 2.11 ± 1.49 logMAR; *P* = 0.025).

**Conclusion:**

An early rise in IOP is a significant risk factor which compromises the visual outcome of patients undergoing diabetic vitrectomy.

An early rise in intraocular pressure (IOP) following vitreoretinal surgery is a well-recognized complication, occurring in 13–40% of cases.[1–3] The cause of an early rise in IOP may either be the underlying predisposition to developing glaucoma or it can be a complication secondary to the vitreoretinal procedure.[4–6]

The role of diabetes mellitus as a significant risk factor for glaucoma remains controversial.[7,8] Although studies do show an increased occurrence of glaucoma following pars plana vitrectomy (PPV) for proliferative diabetic retinopathy (PDR),[3,9] however to our knowledge no study reveals the risk factors leading to a postoperative early rise in IOP in patients of PDR following PPV.

The present study was designed to assess the incidence and risk factors for an early rise in IOP in patients undergoing PPV for the management of PDR.

## Materials and Methods

We conducted a longitudinal prospective study on patients of PDR admitted in the vitreoretinal unit of our institute, who underwent standard 20-gauge 3-port PPV (performed by YRS, RVA, and AK).

Cases with vision of “no perception of light,” coexistent primary or secondary rhegmatogenous retinal detachment, presence of any rubeosis iridis, preoperative glaucoma (defined as IOP greater than 21 mmHg, or glaucomatous changes in the disc in cases where the fundus details were visible), and prior ocular surgery, including cataract surgery, were excluded from the study. Also patients with coexistent disease making survival or availability for regular follow-up visits for at least 6 months unlikely were also excluded from the study. Informed consent was taken from all patients before enrolling them in the study.

Information regarding the patients’ demographic baseline parameters, clinicopathological parameters, past medical and ophthalmic history, ophthalmic findings including best corrected visual acuity (BCVA), slit-lamp biomicroscopy, indirect ophthalmoscopy, and Goldmann applanation tonometry were recorded preoperatively and at follow-up. Data regarding the operative procedures, complications, and findings were also recorded.

All the patients underwent standard 20-gauge 3-port PPV. Additional procedures and surgical techniques were performed as deemed necessary by the operating surgeon; these included membrane segmentation and delamination, fibrovascular frond removal, silicone oil infusion (SOI), and need for lens removal if poor visibility mandated it. Intraoperative vitreous bleed, which was defined as intraoperative bleeding requiring temporarily halting the surgery and controlling the bleed, was controlled using endocautery and by temporarily raising the irrigation bottle height. The surgery was reinitiated only after adequate hemostasis and clearance of hemorrhage.

All patients had a postoperative follow-up of at least 6 months. The postoperative evaluation involved follow-up at day 1, week 1, and months 1, 3, and 6 (final follow-up). The main outcome measures recorded were IOP and BCVA, which was recorded using logMAR charts. An early rise in IOP was defined as IOP ≥ 30 mmHg at day 1.[1,2,13] And consistently elevated IOP was defined as any postoperative IOP of > 21 mmHg during first three consecutive follow-up visits. Appropriate medical or surgical intervention was undertaken to reduce the raised IOP. In order to facilitate statistical analysis, appropriate medical or surgical intervention was defined as successful if an IOP ≤ 21 mmHg was achieved.

The management of raised IOP involved administration of topical β-blockers, α-agonists, and oral and topical carbonic anhydrase inhibitors. Trabeculectomy with antimetabolites or cyclodestructive procedures, with or without silicone oil removal when applicable, were employed when medical treatment failed to control IOP.

All the potential risk factors (preoperative, intraoperative, and postoperative) were recorded and fed into the statistical spreadsheet as discreet or continuous variables and analyzed using SPSS for Windows version 10.0. The independent samples *t*-test was used to compare continuous variables between groups. The χ^2^ -test was used to compare discrete variables. The *P*-values < 0.05 were considered significant.

## Results

A total of 73 eyes of 52 males and 21 females were included in the analysis.

Demographic and clinical characteristics of all patients are summarized in Tables 1 and 2.

**Table 1:** Demographic and clinical parameters of all the cases enrolled in the study

Variables	Mean	SD
Age (years)	52.90	10.49
Age at manifestation of diabetes (years)	39.28	10.13
Duration of diabetes mellitus (years)	13.62	6.53
Glycosylated hemoglobin, HbA1c (%)	7.99	1.39
Pre-operative BCVA (logMAR)	2.43	0.87
Pre-operative IOP (mmHg)	15.71	3.19

BCVA: Best corrected visual acuity, IOP: Intraocular pressure, SD: Standard deviation

**Table 2:** Details of intraoperative events and postoperative intraocular pressure of the cases enrolled in the study

**Variables intraoperative details**	**N**	**Percentage**
Fibrovascular frond removal	38	52.1
Intraoperative iatrogenic breaks	15	20.5
Intraoperative vitreous bleed	27	37.0
Lensectomy	3	4.1
Silicon oil infusion	37	50.7
**Postoperative intraocular pressure (mmHg)**	**Mean**	**SD**
Day 1	21.8	9.8
Week 1	18.3	8.1
Month 1	17.9	9.9
Month 3	16.2	7.8
Final follow-up (6 months)	16.7	6.0

SD: standard deviation

Postoperatively, at day 1, the mean IOP was 21.8 ± 9.8 mmHg and 15 out of 73 cases (20.5%) were found to have an early rise in IOP, i.e., IOP ≥ 30 mmHg at day 1. On subsequent follow-up at week 1, month 1, month 3, and month 6, the mean IOP was 18.3 ± 8.1 mmHg, 17.9 ± 9.9 mmHg, 16.2 ±7.8 mmHg, and 16.7 ± 6.0 mmHg, respectively [Table 2].

Five out of 15 cases with an early rise in IOP developed a consistently raised IOP. There was a statistically significant association between early rise in IOP and consistently raised IOP (*P* = 0.02). Medical therapy was the primary mode of management instituted in all patients with raised IOP. Use of topical and systemic antiglaucoma medications controlled IOP in 12 of 15 eyes. Ten out of these 12 patients responded to topical timolol maleate 0.5% twice daily, topical brimonidine 0.15% three times daily, and/or topical dorzolamide hydrochloride 2.0%, which were continued for a period of 1 month. Other two cases required an additional oral acetazolamide 500–750 mg daily. Both these cases responded to medical management and by 3 months had been weaned off oral acetazolamide but still required the use of topical medication, namely, timolol maleate 0.5% twice daily and topical brimonidine 0.15% three times daily. The remaining three cases with medically uncontrolled glaucoma, eventually developed neovascular glaucoma. Two out of these three cases underwent diode laser contact transscleral cyclophotocoagulation, whereas one patient underwent anterior retinal cryotherapy following which a trabeculectomy with mitomycin-C was done at 6 months of follow-up, finally leading to the control of IOP.

For the purpose of the study, the cases were grouped into two: group A – with an early rise in IOP, i.e., IOP ≥ 30 mmHg at day 1 (15 cases) and group B – those without any early rise in IOP, i.e., IOP < 30 mmHg at day 1 postoperatively (58 cases). Various preoperative and intraoperative risk factors were evaluated for any association with this early elevation in IOP [Table 3]. Preoperative factors like sex, type of diabetes mellitus, previous panretinal photocoagulation (PRP), coexistent vitreous hemorrhage, coexistent macular traction retinal detachment (TrRD), and poor glycemic control (HbA_1_c > 8.0%) were not found significant. However, an early rise in IOP was found to be significantly associated with intraoperative risk factors like fibrovascular frond removal (*P* = 0.003), intraoperative vitreous bleed (*P* = 0.008), and lens removal (*P* = 0.043).

**Table 3:** Chi-square analysis of preoperative and intraoperative variables predicting the early elevation in intraocular pressure

		Intraocular pressure ≥ 30 mmHg		*P*-value
		Yes	No	
Sex	Male	12	40	0.40
	Female	3	18	
Eye side	OD	7	39	0.14
	OS	8	19	
Preop. vitreous hemorrhage	Yes	14	50	0.45
	No	1	8	
Macular traction RD	Yes	10	23	0.06
	No	5	35	
Previous PRP	Yes	10	35	0.65
	No	5	23	
Diabetes mellitus type	Type 1	3	4	0.12
	Type 2	12	54	
Glycosylated hemoglobin, HbA_1_c (%)	Poor control (>8.0 %)	9	21	0.09
	Adequate control (≤8.0%)	6	37	
Fibrovascular frond removal	Yes	13	25	<0.001
	No	2	33	
Membrane dissection over macula	Yes	10	38	0.93
	No	5	20	
Intraoperative iatrogenic breaks	Yes	4	11	0.51
	No	11	47	
Intraoperative vitreous bleed	Yes	10	17	<0.001
	No	5	41	
Lens removal	Yes	2	1	0.04
	No	13	57	
Silicone oil infusion	Yes	10	27	0.17
	No	5	31	

**Figure 1:**
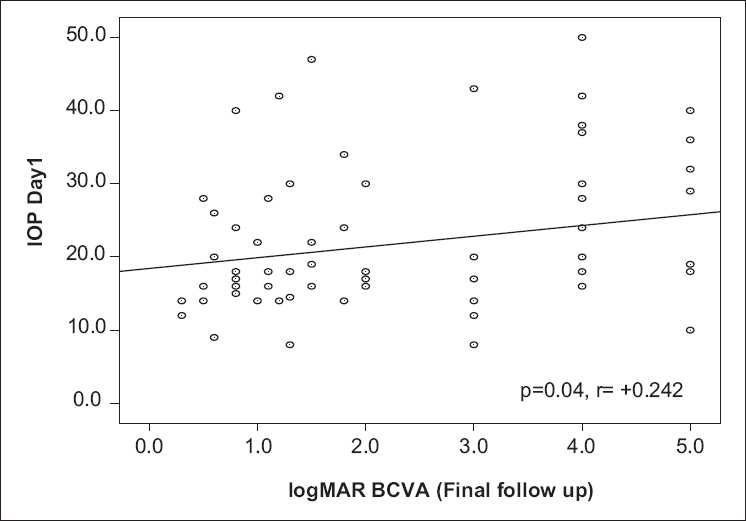
A scattergram showing correlation between mean postoperative intraocular pressure (IOP; mmHg) at day 1 and mean postoperative best corrected visual acuity (logMAR units) at the final follow-up (6 months)

Although the preoperative BCVA values between groups A and B were comparable (2.57 ± 0.93 logMAR vs. 2.39 ± 0.85 logMAR, respectively; *P* = 0.48), there existed a statistically significant difference on comparing the final BCVA values between the two groups (3.11 ± 1.52 logMAR vs. 2.11 ± 1.49 logMAR, respectively; *P* = 0.025). Analysis also revealed that there was a positive correlation between IOP on the first postoperative day and logMAR BCVA at the final (6 months) follow-up (*P* = 0.04, *r* = +0.24) [Fig. 1].

On subgrouping cases into those with SOI (37 cases, 50.7%) and those without SOI (36 cases, 49.3%), the mean of IOP (day 1) was 22.9 ± 10.5 mmHg and 20.76 ± 9.10 mmHg, respectively. This was however not statistically significant (*P* = 0.36). Similar was true for IOP recordings over rest of the follow-up periods, except for IOP at the week 1 follow-up where this difference was found to be statistically significant (*P* = 0.04) [Table 4].

**Table 4:** Comparison of values of intraocular pressure among the cases with and without silicone oil tamponade (*t*-test)

	Mean intraocular pressure (mmHg)		*P*-value
	With silicone oil	Without silicone oil	
Preoperative	15.55 ± 3.00	15.86 ± 3.42	0.67
Postoperative			
Day 1	22.87 ± 10.45	20.76 ± 9.10	0.36
Week 1	20.16 ± 9.60	16.28 ± 5.71	0.04
Month 1	18.35 ± 10.92	17.42 ± 8.90	0.69
Month 3	16.00 ± 8.36	16.31 ± 7.34	0.86
Month 6	16.97 ± 5.54	16.36 ± 6.59	0.67

## Discussion

The present study was undertaken to assess the factors responsible for the early rise in IOP which is a relatively common occurrence after vitreoretinal surgery,[10–12] particularly so in PPV for complications of PDR.[13]

de Corral *et al*. found that diabetes mellitus was not associated with pressure problems,[14] but Ando found that diabetic patients were likely to have a postoperative pressure rise, more so those who were aphakic.[15] Also studies have demonstrated that cases with elevated IOP at day 1 postoperatively are more likely to have consistently raised IOP, i.e., IOP > 21 mmHg beyond 3 weeks of follow-up,[13] which in the present study is represented by postoperative IOP of > 21 mmHg during first three consecutive follow-up visits (visits at day 1, week 1, and month 1). Besides the contradictory findings of the above studies, changes in clinical practice and improvement in instrumentation and skills make extrapolating data from older studies to current clinical practice difficult.

Our study lays emphasis on the postoperative IOP rise in series of cases which underwent PPV for various complications of proliferative diabetic retinopathy with a particular focus on an early rise in IOP. In this study, 20.5 % cases had an early rise in IOP. This is comparable to previous studies that have reported 14.8–35.6% of the cases developing post-PPV early IOP rise.[10–12] As reported in a previous study,[13] this study also revealed that cases with an early rise in IOP are more likely to have a persistent elevation in IOP (*P* = 0.02). This was evident by the fact that 5 out of 15 cases with an early rise in IOP developed a persistent rise in IOP requiring medical or surgical management.

Although none of the preoperative factors was found to contribute to this early IOP rise, it was noted that 10 out of 15 cases with macular traction retinal detachment were found to have an early rise in IOP. However, this finding was not statistically significant (*P* = 0.06). Intraoperative factors which were associated with postvitrectomy early rise in IOP included fibrovascular frond removal (*P* = 0.003), lens removal (*P* = 0.043), and intraoperative vitreous bleed (*P* = 0.008). A further analysis of intraoperative variables showed that 38 out of 73 cases (52.1%) underwent intraoperative fibrovascular frond removal. Out of these 38 cases, 24 cases (63.2%) had intraoperative vitreous bleed, 11 cases (28.9%) had intraoperative break, and 2 cases (5.3%) required intraoperative lens removal due to intraoperative problems in visualizing the fundus. These events themselves suggest complicated diabetic vitrectomy involving more intraoperative manipulations and prolonged surgery; this can affect both the ciliary body and the trabecular meshwork function. In our view, the most plausible reasons leading to an early rise in IOP in postdiabetic vitrectomies are inflammatory and erythroclastic trabecular meshwork obstruction and/or ciliary body edema leading to the relative pupillary block.

The importance of raised IOP leading to a poor functional outcome cannot be overemphasized. A significant positive correlation between final logMAR BCVA and IOP at day 1 (*P* = 0.04, *r* = +0.24) suggested that cases with an early IOP rise are likely to have a poorer visual outcome. This is due to the fact that an early rise of IOP is more likely to have persistently raised IOP requiring medical or surgical therapy, as mentioned previously, hence leading to poorer BCVA at 6 months of follow-up.

SOI was not found to be significantly associated with the early IOP rise (*P* = 0.17). This might be attributed to the fact that none of the cases at postoperative day 1 had silicone oil overfill. This was primarily due to adequate care taken to keep the closing pressure in each case below 10 mmHg during air–silicone oil exchange. However, mean IOP values at week 1 postoperatively were found to be significantly greater in the case with silicone oil tamponade as compared to cases without silicone oil tamponade (*P* = 0.04). This signified that the cases with SOI showed a more gradual drop in IOP postoperatively, taking a longer time in returning to preoperative values as compared to cases without SOI [Table 4].

We summarize that an early rise in IOP after diabetic vitrectomy is more likely to be seen in cases with prolonged and complex intraoperative maneuvers, and that these cases with an early rise in IOP may be associated with a poorer visual outcome which in part is related to the fact that these cases are more likely to have a persistently raised IOP.
